# Minimally invasive surgery for colorectal cancer remains underutilized in Germany despite its nationwide application over the last decade

**DOI:** 10.1038/s41598-018-33510-y

**Published:** 2018-10-11

**Authors:** Tarik Ghadban, Matthias Reeh, Maximilian Bockhorn, Asmus Heumann, Rainer Grotelueschen, Kai Bachmann, Jakob R. Izbicki, Daniel R. Perez

**Affiliations:** Department of General, Visceral and Thoracic Surgery, University Medical Center Hamburg-Eppendorf, Martinistrasse 52, 20246 Hamburg, Germany

## Abstract

Minimally invasive surgery (MIS) has superior short-term outcomes than open surgery (OS) for colorectal cancer (CRC). However, a nationwide dataset has not been analysed to confirm these findings. We evaluated the distribution and outcomes of MIS for CRC from 2005 to 2015; all in-patients with CRC surgery procedure codes were identified from hospital data, which are entered into the nationwide diagnosis-related group database and forwarded anonymised to the Federal Bureau of Statistics. We determined absolute MIS, morbidity, and mortality rates for specific sub-categories, including procedure type. We identified 345,913 in-patient files. The MIS rate increased from 6.4% (*n* = 2366; 2005) to 28.5% (*n* = 8363; 2015), with the highest rates for sigmoid colon (38%) and rectal (39%) resections. The overall conversion rate was 14.4%, without noticeable improvement over time. International Classification of Disease codes related to postoperative complications were documented more frequently after OS than after MIS. OS was associated with a higher mortality rate (4.7%) than MIS (1.8%) (P < 0.001), even after stratifying patients according to the resection site. Use of MIS remains low in Germany compared with that in other European countries. Underutilization of MIS has to be addressed in the future by promoting structured training programs and standardization of laparoscopic surgery.

## Introduction

In Germany, over 60,000 cases of colorectal cancer (CRC) are diagnosed each year. CRC is the third most common cancer in both men and women, and 25,000 patients die from CRC each year^1^.

Surgery is a cornerstone of treatment for patients with CRC^2^, and it has traditionally been performed through an open abdominal incision. Conventional open surgery is associated with significant morbidity and long periods of convalescence. In 1991, Jacobs *et al*. reported the first laparoscopic colectomy^3^. Over the last decade, surgical treatment has improved considerably in relation to preoperative assessments, laparoscopic instruments, surgical techniques, intraoperative patient management, and postoperative care.

Several international randomised clinical trials have demonstrated comparable oncological outcomes and better short-term outcomes for minimally invasive surgery compared with those for open surgery^4–15^.

Randomised trials have strict inclusion criteria and those comparing open surgery with minimally invasive surgery are often performed at centres with high levels of expertise in minimally invasive surgery. Hence, the data reported from these studies might not be applicable to many hospitals, because of infrastructure shortages or a lack of staff who are suitably skilled in laparoscopy. However, many nationwide and registry analyses have reproduced the outcomes from the randomised controlled trials within large cohorts of patients who were managed in real-world clinical settings^16–20^.

In 2015, about 30,000 surgical procedures for CRC were undertaken in Germany, and the published data suggest low morbidity and mortality rates^21^. However, these data were generated by single- or multi-centre studies and they are biased, because these centres were experienced in minimally invasive surgery and had high caseloads that formed the basis of their published data, whereas less experienced centres do not tend to publish their results. Given that a large proportion of patients do not undergo surgery in high-volume centres, the published data may lead to misinterpretations of the outcomes of surgery for CRC in Germany.

Since 2004, German hospitals’ remuneration for in-patient treatment has been regulated through the diagnosis-related group system (DRG). Data relating to every in-patient hospital stay, which includes the diagnosis and procedure codes, are entered into the nationwide diagnosis-related group database, and the staff at the diagnosis-related group data centre forward the anonymised data to the Federal Bureau of Statistics. Individuals’ hospitalisation data are available for research purposes within the context of the regulations that surround confidentiality. The hospitalisation data are anonymised; hence, patients who are hospitalised more than once during a study period cannot be re-identified. Federal law allows these anonymised data to be used for scientific purposes without an ethical review. Many highly influential studies have been published that are based on diagnosis-related group data^22–25^.

However, a nationwide dataset has not been analysed to confirm that minimally invasive surgery has superior short-term outcomes than open surgery for CRC. The aim of this study was to evaluate the distribution and outcomes of minimally invasive surgery for CRC over an 11-year period using a comprehensive German dataset.

## Results

### Surgical procedures

**Figure 1:**
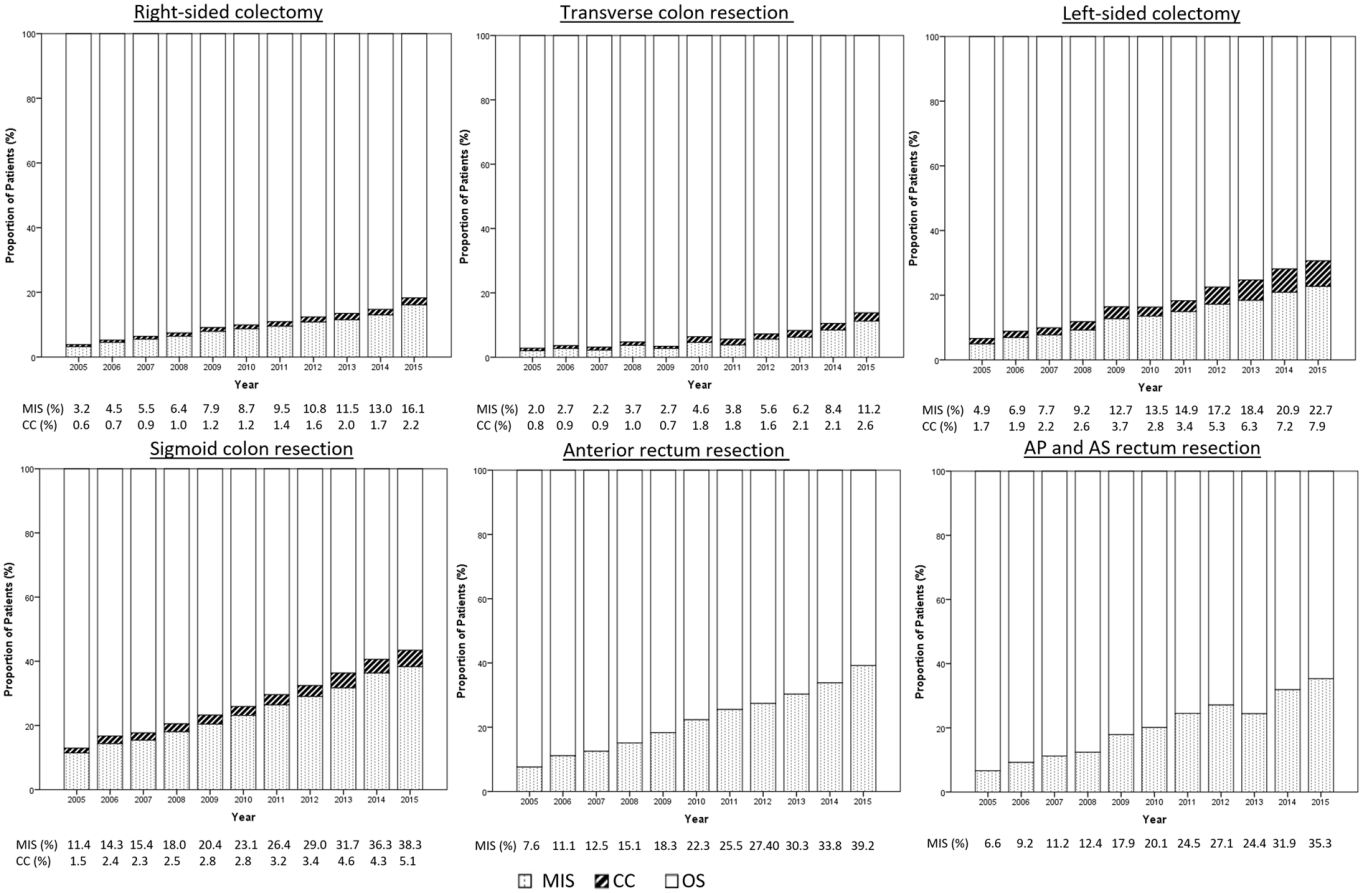
Distributions of the different surgical approaches according to the resection site. AP: abdominoperineal, AS: abdominosacral, MIS: minimally invasive surgery, CC: converted cases, OS: open surgery.

A total of 345,913 elective procedures for CRC were identified that included 211,157 (61.0%) colonic and 134,756 (39.0%) rectal resections. Minimally invasive surgery was completed in 55,323 (16.0%) patients. The proportion of cases that underwent minimally invasive surgery increased continuously. In 2005, 6.4% of the cases underwent minimally invasive surgery and, in 2015, 28.5% of the cases underwent minimally invasive surgery. Regarding the trends in the surgical modalities, there was a 22.1% decrease in open surgery and a 4.5-fold increase in minimally invasive surgery. Minimally invasive surgery was undertaken most frequently for sigmoid colon and anterior rectal resections, and its use for right-sided colectomies and transverse colon resections remained low. The highest rates for laparoscopic surgery of the ascending and transverse colon were 16.1% and 11.2%, respectively, and these occurred in 2015. Figure 1 shows the distributions of the use of minimally invasive surgery for the different CRC locations. A total of 31,481 colon cancer cases was scheduled to undergo minimally invasive surgery, and, of these, 4,532 (14.4%) cases were converted to open surgery.

### Mortality

The overall in-hospital mortality rates for open and minimally invasive surgery were 4.7% and 1.8%, respectively, (P < 0.001). In relation to all of the surgical procedures, minimally invasive *surgery was associated with significantly lower mortality rates (P < 0.001) (*Table 1*;* Supplementary Table S1*). The mortality rates associated with laparoscopic right-sided* colectomies, left-sided colectomies, sigmoid resections, and anterior rectum resections were lower in most of the years analysed compared with the mortality rates for similar open surgical procedures. The annual mortality rates associated with laparoscopic transverse colon resections, abdomino-perineal, and abdomino-sacral rectum resections were not lower than the annual mortality rates for similar open surgical procedures.

**Table 1:** Mortality rates in percent for the different procedures of procedures undertaken. OS: open surgery, MIS: minimally invasive surgery.

Procedure		2005	2006	2007	2008	2009	2010	2011	2012	2013	2014	2015	Overall
Right-sided colectomy	OS	5.0	5.3	5.3	5.2	5.0	5.3	4.9	4.7	4.6	4.8	4.3	5.0
	MIS	3.1	1.6	2.3	3.2	2.9	2.0	2.4	2.2	2.0	1.5	2.2	2.2
	p-value	0.102	<0.001	0.002	0.021	0.007	<0.001	0.001	<0.001	<0.001	<0.001	<0.001	<0.001
Transverse colon resection	OS	8.2	7.1	8.8	7.7	5.7	7.4	8.2	7.5	6.1	7.5	7.5	7.4
	MIS	7.2	0	0	4.5	0	0	4.3	3.1	2.9	0	1.8	1.9
	p-value	0.697	0.145	0.154	0.527	0.210	0.061	0.421	0.277	0.346	0.02	0.06	<0.001
Left-sided colectomy	OS	5.2	4.1	5.0	4.2	5.4	5.4	4.5	5.1	5.3	4.8	5.5	4.9
	MIS	1.5	2.6	0.6	0.6	0.4	1.8	1.2	1.7	2.1	1.5	1.3	1.4–1.5
	p-value	0.024	0.249	0.002	0.002	<0.001	0.002	0.002	0.001	0.002	<0.001	<0.001	<0.001
Sigmoid colon resection	OS	4.6	5.2	5.6	5.3	5.3	5.9	6.0	5.1	4.8	5.2	6.4	5.3
	MIS	1.0	1.2	0.8	1.5	1.1	1.6	1.2	1.2	1.2	1.4	2.0	1.3
	p-value	<0.001	<0.001	<0.001	<0.001	<0.001	<0.001	<0.001	<0.001	<0.001	<0.001	<0.001	<0.001
Anterior rectum resection	OS	4.0	3.7	3.8	3.8	3.5	4.1	3.6	3.4	3.4	3.3	3.7	3.7
	MIS	2.3	1.0	1.8	2.4	1.8	2.0	2.0	1.4	1.8	1.5	1.8	1.8
	p-value	0.016	<0.001	0.001	0.007	<0.001	<0.001	<0.001	<0.001	<0.001	<0.001	<0.001	<0.001
Abdominoperineal and abdominosacral rectum resection	OS	3.5	4.4	4.0	4.1	4.3	3.8	4.4	3.3	4.6	3.8	3.3	4.0
	MIS	3.0	2.7	3.1	3.7	1.4	2.4	2.7	2.2	2.5	3.4	2.4	2.6–2.7
	p-value	0.670	0.163	0.436	0.684	0.002	0.104	0.070	0.1511	0.023	0.666	0.226	<0.001

### Morbidity

**Figure 2:**
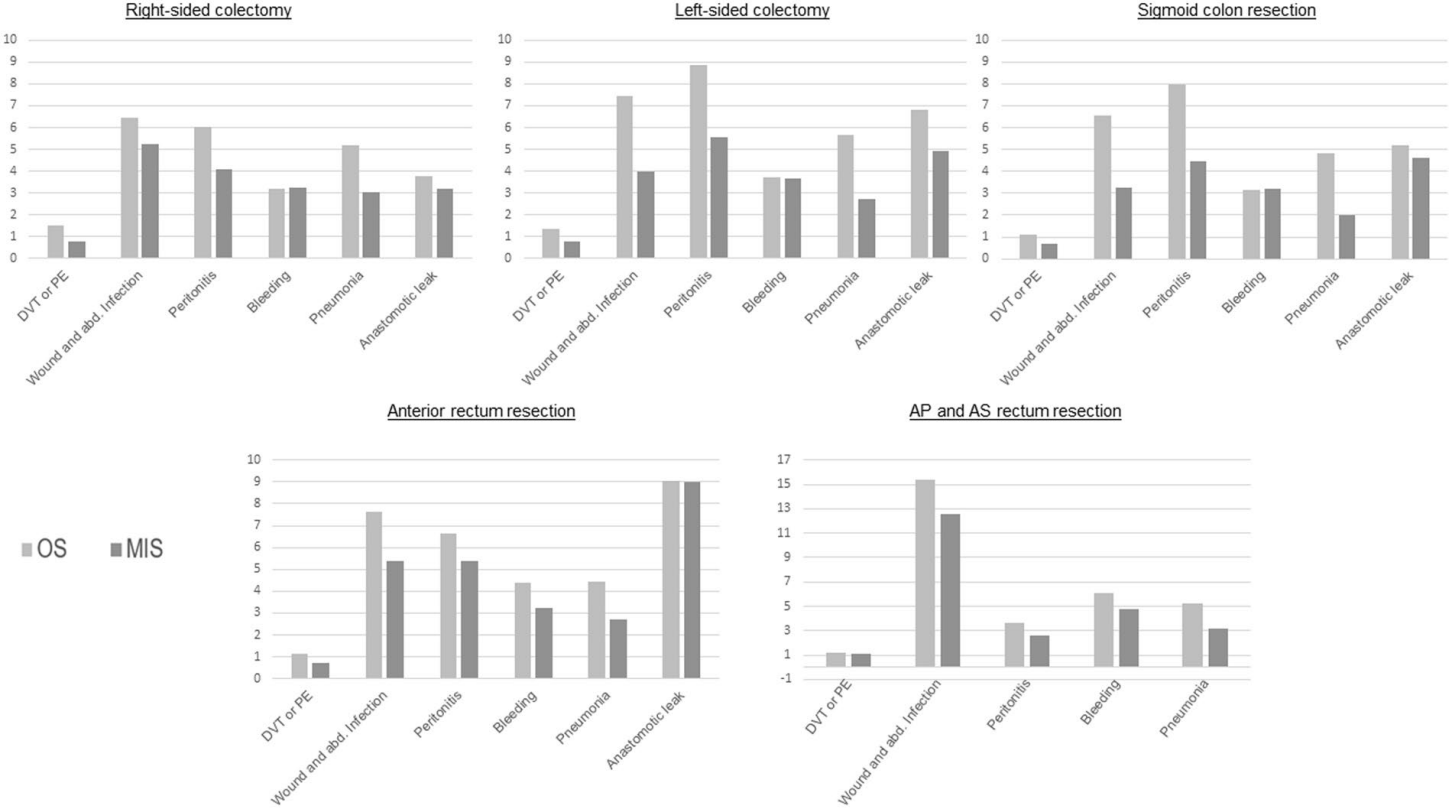
Morbidity rates in percent for the different procedures. OS: open surgery, MIS: minimally invasive surgery, AP: abdominoperineal, AS: abdominosacral, DVT: deep vein thrombosis, PE: pulmonary embolism, abd.: abdominal.

We analysed the rates for deep vein thrombosis, pulmonary embolism, wound and abdominal infections, bleeding, and pneumonia and those for anastomotic leaks in association with right-sided colectomies, left-sided colectomies, sigmoid resections, anterior rectal resections, and abdomino-perineal and abdomino-sacral rectum resections. The morbidities associated with minimally invasive surgery were similar or less frequent compared with those associated with open surgery. The pneumonia, wound and abdominal infection, and postoperative bleeding rates for patients who had undergone minimally invasive surgery tended to be significantly lower in association with all of the procedures and over the whole study period than those for patients who had undergone open surgery (Fig. 2*;* Supplementary Table S2). Given the low number of patients who underwent transverse colon resections, we did not analyse the morbidity associated with minimally invasive surgery and open surgery for this procedure.

### Hospital stays

The median hospital stays of patients who underwent laparoscopic surgery were shorter for all of the procedures than those of patients who underwent open surgery. While the median hospital stay after minimally invasive colonic resections ranged between 10 and 15 days, it was between 15 and 19 days after open surgery when considering the annual rates. In anterior rectal resections, hospital stay was between 15 and 18 days after minimally invasive surgery and between 11 and 16 days after open surgery. Abdomino-perineal and abdomino-sacral rectum resections had a median hospital stay between 18 and 23 days for minimally invasive procedure and between 15 and 23 days for open procedure.

### Conversion rates

The overall conversion rate was 14.4%, and the conversion rates were 13.1% for right-sided colectomies, 23.9% for transverse colon resections, 21.4% for left-sided colectomies, and 11.8% for sigmoid colon resections. The conversion rates ranged between 18.4% and 32.7% for transverse colon resections and left-sided colectomies and between 10.4% and 16.7% for right-sided colectomies and sigmoid colon resections when considering the annual rates. Regarding rectal surgery, there were no specific codes for the cases that were converted to open surgery; therefore, these rates cannot be reported.

## Discussion

Colorectal resections are common procedures, and previous findings show low mortality and morbidity rates^19,26^. The development of minimally invasive surgery in recent years has led to steady increases in the number of laparoscopic colorectal resections^17,19,20^. The findings from several randomised trials have demonstrated that the long-term outcomes associated with laparoscopic resections are comparable with those associated with open surgical resections, and that laparoscopic resections seem to be superior than open resections in terms of short-term outcomes^4–9,13^. These findings have increasingly propagated the use of minimally invasive surgery as the standard procedure.

The recently reported findings from the ACOSOG Z6051^27^ and ALaCaRT^28^ trials, which showed that laparoscopy failed to show non-inferiority compared with open surgery in relation to the pathological assessments, contradict the data from the COREAN^11^ and COLOR II^12^ trials that were unable to demonstrate any differences between laparoscopic surgery and open surgery in relation to the short- and long-term outcomes or the quality of the oncological resections. Short- and long-term oncological outcomes from the ACOSOG Z6051 and ALaCaRT trials have not been reported.

Our data show that the proportion of laparoscopic resections performed increased continuously between 2005 and 2015. However, open surgery remained the main resection method. Indeed, 75.0% and 71.5% of the procedures undertaken used conventional open surgical techniques in 2014 and 2015, respectively. Comparing the surgical modality trends, there was a 22.1% decrease in open surgery and a 4.5-fold increase in laparoscopic surgery. In 2015, only 16.1% and 11.2% of the right-sided colectomies and transverse colon resections, respectively, were performed laparoscopically, but sigmoid colon and anterior rectal resections were more regularly performed laparoscopically at rates of 38.3% and 39.2%, respectively, which are lower than those reported from other countries. A Norwegian analysis demonstrated an increase in the rate of laparoscopic resections from 15.9% in 2007 to 35.7% in 2010 for left-sided, right-sided, and sigmoid tumours, and an overall laparoscopic resection rate of 27% between 2007 and 2010^29^. Our study’s findings show that the laparoscopic resection rates during the same time span and for the same indications were only 8.7% in 2007 and 12.9% in 2010, and that the overall laparoscopic resection rate was only 10.9%. The findings from a French analysis showed a laparoscopic resection rate of 26% between 2006 and 2008^26^.

The overall conversion rate in the current study was 14.4%, which is lower than the conversion rates determined from the COST (21%) and CLASSIC (19%) trials^5,6^, and it is comparable with the conversion rate reported by Stormark *et al*. (14.5%)^29^. Clear differences in the conversion rates were found among the different procedures; hence, the conversion rates for right-sided colectomies and sigmoid colon resections were lower than those for transverse colon resections and left-sided colectomies. There was no clear-cut improvement in the conversion rate over time, which concurs with previously published data^29^. It is possible that the increase in the proportion of laparoscopic resections performed over the years is indicative of the extension of the indications for which the laparoscopic approach can be used compared with the limited number of indications for which laparoscopy could be used during the earlier years; this has occurred as the level of expertise in laparoscopy has increased.

The mortality rates did not differ over the years, and laparoscopic resections were associated with significantly lower mortality rates than conventional resections (P < 0.001). The mortality rates were comparable across all of the procedures, except for those associated with the transverse colon resections that were performed using open surgery, which were much higher than the mortality rates associated with the other resections that were performed using open surgery. The improved mortality rates associated with laparoscopic surgery demonstrated in the current investigation are consistent with those reported by previous investigators^19,26^. Our study’s hospital mortality rates were higher than those recently published by Lee *et al*., who demonstrated hospital mortality rates of 2.39% for open and 0.95% for laparoscopic resections^19^, and they were lower than those published by Panis *et al*. who demonstrated hospital mortality rates of 2% after laparoscopy and 6% after laparotomy^26^. Furthermore, the present study is the first to analyse the hospital mortality rates with respect to different resection sites.

Regarding the morbidity rates, laparoscopic surgery was equal or superior to open surgery with respect to all of the aspects of morbidity investigated, including the rates of pneumonia and wound and abdominal infections, all of which were significantly lower when the procedures were performed laparoscopically. The deep vein thrombosis and pulmonary embolism rates were also lower, and the low rates of these particular complications could explain the absence of a significant difference.

The anastomotic leak rates associated with the colectomies demonstrated the superiority of a laparoscopic approach that was significant for right-sided colectomies between 2014 and 2015 and for left-sided colectomies between 2008 and 2015. We were unable to clearly demonstrate the superiority of a laparoscopic approach for rectal resections between 2011 and 2013; hence, with the exception of the rectal resections, these findings are comparable with those recently published by Murray *et al*.^30^.

To our knowledge, this is the first nationwide investigation of the approaches used for colorectal surgery and their mortality and morbidity rates in Germany. Although it is likely that the present study portrays a representative depiction of colorectal surgery in Germany, it does have some limitations. The anonymization of the diagnosis-related group data rule out any validation of the data that could be achieved by comparing the assigned codes with representative samples of the patients’ case notes. Diagnosis-related group validation studies at particular hospitals would not be of any benefit, because they would not be representative of the population as a whole and routine documentation in hospitals cannot necessarily be considered the gold standard for validation. Further limitations are missing covariates, including patient, surgeon, and hospital factors, on which the decision to pursue a minimally invasive approach particularly depends. Furthermore, incorrect coding may influence our data; usually coding is controlled through several instances in the hospital because financial remuneration depends on coding. Therefore, the plausibility of our findings has to be critically considered from the perspectives of the existing clinical and epidemiological knowledge and other data.

A major strength of this study is associated with the completeness of the database. Our analysis was based on data from all hospital-based visits and, it is, therefore, not affected by selection biases originating from the selective inclusion of specific hospitals, health insurance systems, or age groups. Therefore, our data represent the parent population rather than a statistical sample, and the results are true values as opposed to estimates. Consequently, neither statistical hypothesis testing to exclude sampling errors nor calculated measurements of probability distributions were necessary. Moreover, the data were unaffected by the hospitals’ own determinations or self-reporting.

In conclusion, our data demonstrate the superiority of a laparoscopic approach for CRC surgery. This study’s findings are comparable with published data that describe the superiority of the laparoscopic approach. Our findings also clearly demonstrate increases in the use of the laparoscopic approach in Germany over the time span investigated; however, the rate remains low. National health policies should aim at promoting training programs and standardisation of laparoscopic surgery in the future.

## Methods

### Data

We undertook a controlled remote analysis of in-patient data that had been entered into the nationwide diagnosis-related group database from 2005 to 2015, which were provided by the Research Data Centers of the Federal Statistical Office and the Statistical Offices of the Länder. The diagnoses and procedures were identified using the German adaptation of the International Classification of Diseases (ICD-10-GM) and the German procedure coding system (OPS) that corresponded to each year.

### Case definitions, types of surgery, and principal diagnoses

We included every in-patient case that had a procedure code for a non-emergency colorectal resection and CRC as the primary diagnosis. The pertinent procedures included laparoscopic, converted, and open right-sided colectomies, transverse colon resections, left-sided colectomies, and sigmoid colon resections. Furthermore, laparoscopic and open anterior rectal resections, abdomino-perineal rectal resections, and abdomino-sacral rectal resections were included. The procedure coding system does not contain specific codes for cases that were converted during rectal surgery. Rectal resections are sub-grouped into high and low resections, and those with perianal anastomoses. Those patients who underwent laparoscopic surgery and were converted to open surgery were classified as having undergone open surgery. Emergency procedures and colectomies for benign diagnoses were excluded from the analysis. The International Classification of Disease codes used were C18–20 for the main diagnoses, and the German procedure codes used were 5-455.01-7, 5-455.51-7, 5-456.01-7, 5-457.01-7, 5-484.31-9, 5-484.51-9, 5-484.61-9, 5-485.01-2, and 5-485.21-2.

### Mortality

For each case, DRG data include the dismissal reason with a specific code for in-hospital death. Therefore, our reported mortality represents in-hospital mortality.

### Morbidities

The patients’ morbidities were analysed using secondary diagnosis and procedure codes that indicated complicated courses of treatment, and the codes used were I26, T81.0, T81.1, T81.3, T81.4, I80.2, K65, J1, and K91.83.

### Statistical analyses

We analysed the data descriptively. Simple linear regression models, including the method of weighted least squares based on the number of observations, were used to assess temporal trends. A value of P < 5% was considered to indicate the presence of a statistically significant linear trend during the years that were included in the analysis. The statistical analyses were conducted using IBM^®^SPSS^®^ software for Windows, version 22.0 (IBM Corporation, Armonk, NY, USA).

## Electronic supplementary material

Supplementary Tables

## Data Availability

The datasets generated during and/or analysed during the current study are available from the corresponding author on reasonable request.
