# Supplementary material for: Minimally invasive surgery for colorectal cancer remains underutilized in Germany despite its nationwide application over the last decade
Source: Sci Rep. 2018 Oct 11;8:15146. doi: 10.1038/s41598-018-33510-y (PMC6181957; doi:10.1038/s41598-018-33510-y)
Supplement: Supplementary file 1 — Supplementary Tables [file 41598_2018_33510_MOESM1_ESM.pdf]

Minimally invasive surgery for colorectal cancer remains underutilized in Germany despite its nationwide application over the last decade

Tarik Ghadban†, Matthias Reeh†, Maximilian Bockhorn, Asmus Heumann, Rainer Grotelueschen, Kai Bachmann, Jakob R. Izbicki and Daniel R. Perez*

† These authors contributed equally to this work

Supplementary Table S1: Mortality rates for the different procedures with the absolute numbers of procedures undertaken. OS: open surgery, MIS: minimally invasive surgery

Procedure		2005	2006	2007	2008	2009	2010	2011	2012	2013	2014	2015	Overall
Right-sided colectomy	OS	10771	10544	9633	9648	9586	9549	9348	8899	9172	8961	8613	104724
	Mortality (%)	5.0	5.3	5.3	5.2	5.0	5.3	4.9	4.7	4.6	4.8	4.3	5.0
	MIS	354	495	557	665	826	909	980	1083	1188	1337	1656	10050
	Mortality (%)	3.1	1.6	2.3	3.2	2.9	2.0	2.4	2.2	2.0	1.5	2.2	2.2
	p-value	0.102	<0.001	0.002	0.021	0.007	<0.001	0.001	<0.001	<0.001	<0.001	<0.001	<0.001
Transverse colon resection	OS	1030	1018	929	861	929	919	898	804	802	732	650	9572
	Mortality (%)	8.2	7.1	8.8	7.7	5.7	7.4	8.2	7.5	6.1	7.5	7.5	7.4
	MIS	21	28	21	33	26	44	35	48	53	67	82	458
	Mortality (%)	7.2	0	0	4.5	0	0	4.3	3.1	2.9	0	1.8	1.9
	p-value	0.697	0.145	0.154	0.527	0.210	0.061	0.421	0.277	0.346	0.02	0.06	<0.001
Left-sided colectomy	OS	3803	3596	3144	2948	2699	2521	2377	2276	2289	2187	1970	29810
	Mortality (%)	5.2	4.1	5.0	4.2	5.4	5.4	4.5	5.1	5.3	4.8	5.5	4.9
	MIS	195	266	261	298	392	395	416	472	517	577	578	4367
	Mortality (%)	1.5	2.6	0.6	0.6	0.4	1.8	1.2	1.7	2.1	1.5	1.3	1.4-1.5
	p-value	0.024	0.249	0.002	0.002	<0.001	0.002	0.002	0.001	0.002	<0.001	<0.001	<0.001
Sigmoid colon resection	OS	6118	5588	4700	4179	3644	3228	2875	2664	2701	2490	2344	40531
	Mortality (%)	4.6	5.2	5.6	5.3	5.3	5.9	6.0	5.1	4.8	5.2	6.4	5.3
	MIS	788	931	856	918	936	970	1031	1087	1254	1421	1453	11645
	Mortality (%)	1.0	1.2	0.8	1.5	1.1	1.6	1.2	1.2	1.2	1.4	2.0	1.3
	p-value	<0.001	<0.001	<0.001	<0.001	<0.001	<0.001	<0.001	<0.001	<0.001	<0.001	<0.001	<0.001
Anterior rectum resection	OS	9627	9200	8025	7611	7664	7146	6838	6500	7072	6552	5913	82148
	Mortality (%)	4.0	3.7	3.8	3.8	3.5	4.1	3.6	3.4	3.4	3.3	3.7	3.7
	MIS	790	1144	1148	1358	1711	2049	2340	2455	3080	3349	3806	23230
	Mortality (%)	2.3	1.0	1.8	2.4	1.8	2.0	2.0	1.4	1.8	1.5	1.8	1.8
	p-value	0.016	<0.001	0.001	0.007	<0.001	<0.001	<0.001	<0.001	<0.001	<0.001	<0.001	<0.001
Abdominoperineal and abdominosacral rectum resection	OS	3079	2955	2575	2418	2253	2030	1862	1809	1811	1570	1443	23805
	Mortality (%)	3.5	4.4	4.0	4.1	4.3	3.8	4.4	3.3	4.6	3.8	3.3	4.0
	MIS	218	300	325	341	492	510	604	673	586	736	788	5573
	Mortality (%)	3.0	2.7	3.1	3.7	1.4	2.4	2.7	2.2	2.5	3.4	2.4	2.6-2.7
	p-value	0.670	0.163	0.436	0.684	0.002	0.104	0.070	0.1511	0.023	0.666	0.226	<0.001

Supplementary Table S2: Morbidity rates in percent for the different procedures. *indicates P < 0.05 for minimally invasive surgery compared with open surgery. OS: open surgery, MIS: minimally invasive surgery, DVT: deep vein thrombosis, PE: pulmonary embolism

Procedure		2005-2007		2008-2010		2011-2013		2014-2015	
		OS	MIS	OS	MIS	OS	MIS	OS	MIS
Right-sided colectomy	Total cases	30948	1406	28783	2400	27419	3251	17574	2993
	DVT or PE	1.4	0.2-0.4*	1.5	0.5-0.7*	1.7	1.0*	1.6	0.9*
	Wound/abdominal Infection	5.5	5.5	6.7	6.0	6.9	5.3*	7.1	4.5*
	Peritonitis	5.0	3.4*	5.8	4.3*	6.7	3.9*	7.2	4.5*
	Bleeding	2.9	3.0	3.2	3.0	3.1	3.1	3.8	3.8
	Pneumonia	4.6	2.7*	4.9	2.6*	5.5	3.2*	6.2	3.4*
	Anastomotic leak	3.1	2.3	3.9	3.1	4.2	3.5	4.1	3.3*
Left-sided colectomy	Total cases	10543	722	8168	1085	6942	1405	4157	1155
	DVT or PE	1.0	0.7-1.4	1.3	0.6-0.9	1.6	0.6-0.7*	2.0	0.5-1.0*
	Wound/abdominal Infection	6.3	3.7*	7.5	4.6*	8.2	3.8*	8.9	3.8*
	Peritonitis	6.5	4.3*	8.7	5.4*	10.5	6.5*	12.3	5.3*
	Bleeding	3.2	3.3	3.4	3.4	4.1	4.1	4.9	3.7
	Pneumonia	5.1	2.4*	5.6	2.4*	5.9	2.8*	6.8	3.1*
	Anastomotic leak	5.2	4.6	6.8	5.2*	7.8	4.8*	9.1	5.0*
Sigmoid colon resection	Total cases	16406	2575	11051	2824	8240	3372	4834	2874
	DVT or PE	1.0	0.5-0.6*	1.1	0.6-0.7	1.2	0.7-0.8*	1.4	0.7*
	Wound/abdominal Infection	5.6	2.8*	6.7	3.3*	7.2	3.2*	8.2	3.6*
	Peritonitis	6.2	2.7*	7.9	4.3*	10.2	4.7*	10.5	6.0*
	Bleeding	2.8	2.8	3.1	3.0	3.4	3.3	4.2	3.7
	Pneumonia	4.1	2.1*	4.7	2.0*	5.4	1.8*	6.4	2.1*
	Anastomotic leak	3.9	3.7	5.3	3.9*	6.6	4.9*	6.8	5.7
Anterior rectum resection	Total cases	26852	3082	22421	5118	20410	7875	12465	7155
	DVT or PE	1.0-1.1	0.6-1.0	1.0	0.8-1.0	1.2	0.7*	1.4	0.6*
	Wound/abdominal Infection	6.6	6.0	7.8	5.4*	8.1	5.4*	8.8	5.1*
	Peritonitis	5.3	5.0-5.1	6.5	3.0*	7.2	6.0*	8.8	6.5*
	Bleeding	4.3	3.7	4.3	2.2*	4.3	3.5*	4.9	3.5*
	Pneumonia	4.2	2.6*	4.1	1.6*	4.6	3.0*	5.3	3.3*
	Anastomotic leak	7.6	7.5	9.0	8.4	10.1	9.0*	10.4	10.0
Abdominoperineal and abdominosacral rectum resection	Total cases	8609	843	6701	1343	5482	1863	3013	1524
	DVT or PE	0.9-1.0	0.8-1.3	1.2-1.3	0.6-0.9	1.2-1.3	1.2-1.4	1.6	1.0-1.1
	Wound/abdominal Infection	12.8	10.0*	15.4	13.2*	17.6	12.7*	18.6	13.3*
	Peritonitis	2.6	1.9	3.4	2.3-2.4	4.2	2.6-2.7*	6.1	3.3*
	Bleeding	6.2	5.5-5.7	6.0	5.6-5.7	6.1	4.4-4.5*	5.6	4.0*
	Pneumonia	4.4	3.4-3.7	4.8	2.5*	5.9	2.7-2.8*	7.1	3.9*
